# Prepulse inhibition vs cognitive modulation of the hand-blink reflex

**DOI:** 10.1038/s41598-021-84241-6

**Published:** 2021-02-25

**Authors:** Viviana Versace, Stefania Campostrini, Luca Sebastianelli, Leopold Saltuari, Josep Valls-Solé, Markus Kofler

**Affiliations:** 1 Department of Neurorehabilitation, Hospital of Vipiteno-Sterzing (SABES-ASDAA), Margarethenstr. 24, 39049 Vipiteno-Sterzing, BZ Italy; 2 IDIBAPS (Institut d’Investigació August Pi i Sunyer), Facultat de Medicina, University of Barcelona, Barcelona, Spain; 3 Department of Neurology, Hochzirl Hospital, Zirl, Austria

**Keywords:** Reflexes, Neural circuits

## Abstract

The excitability of brainstem circuitries mediating defensive blinking in response to abrupt sensory inputs is continuously modulated by cortical areas, e.g., the hand-blink reflex (HBR), elicited by intense electrical median nerve stimulation, is enhanced when the stimulated hand is close to the face, with the behavioural purpose to optimize self-protection from increased threat. Here we investigated whether such cortically mediated HBR facilitation can be influenced by prepulse inhibition (PPI), which is known to occur entirely at the subcortical level. Twenty healthy volunteers underwent HBR recordings in five experimental conditions. In *conditions 1 and 2*, the stimulated hand was held either near (1) or far (2) from the face, respectively. In *conditions 3 and 4*, stimulation of the hand near the face was preceded by a peri-liminal prepulse to the index finger of the contralateral hand held either near (3) or far from the face (4). In *condition 5*, participants self-triggered the stimulus eliciting the HBR. We observed a reproducible HBR in 14 out of 20 participants and measured onset latency and area of the HBR in orbicularis oculi muscles bilaterally. HBR area decreased and latency increased in *condition 2* relative to *condition 1*; HBR area decreased and latency increased markedly in *condition 3*, and somewhat less in *condition 4*, relative to *conditions 1 and 2;* self-stimulation (*condition 5*) also suppressed HBRs, but less than prepulses. These findings indicate that PPI of the HBR is more robust than the cognitive modulation exerted by top-down cortical projections. Possibly, an attentional shift to a prepulse may serve to reduce blinking in response to perturbation when it is convenient, in a given situation, not to interrupt ongoing visual processing.

## Introduction

Rapid eyelid closure in response to abrupt and intense environmental stimuli is a prototypical defensive behaviour. This blink reflex, which can be elicited by stimuli of different sensory modalities, is mediated by specific circuits located in the brainstem^1^. Indeed, a variety of afferent inputs converge on brainstem interneurons before activating a selected group of motoneurons in the facial nucleus^2^. Although the most frequently used somatosensory stimulus for both clinical and research purposes is an electrical pulse to the supraorbital nerve, stimulation of non-trigeminal peripheral afferents may also elicit responses in the orbicularis oculi muscle (OOc)^3,4^. In the specific case of median nerve stimulation, Sambo et al.^5^ coined the term “hand-blink reflex” (HBR). They described notable HBR augmentation when the stimulated hand is close to the face as compared to when it is kept farther away from the face. Such enhancement allowed for identification of a potentiated HBR response field around the face, a finding initially interpreted as evidence of a ´defensive peripersonal space´ surrounding the face^5–7^. Numerous publications have since comprehensively characterized the fascinating cognitive tuning of the HBR^5,6,8–14^. Enhancement of the blink reflex in relation to stimuli delivered at a critical distance to the face does not seem to be limited to the HBR, as it may also occur with the trigeminal blink reflex (TBR), when elicited in a particularly threatening context. Interestingly, however, the enhancement was overruled by previous knowledge about the impending stimulus, including self-stimulation^15^.

The magnitude of protective blinking is continuously tuned according to the behavioural relevance of environmental stimuli based on continuous cortically mediated assessment.

A peri-liminal stimulus (prepulse) is known to inhibit the reflex response that would otherwise be elicited by a subsequent suprathreshold stimulus (pulse), a phenomenon called prepulse inhibition (PPI). This polysensory and cross-modal interaction has so far been demonstrated mostly for somatosensory and acoustic prepulses inhibiting trigeminal and auditory blink reflexes^16–20^. PPI mediating circuits are located within the brainstem and include caudal pontine reticular nucleus and pedunculopontine nucleus (for review see^21^).

PPI reflects the pre-conscious ability of the nervous system to filter external (sensory) information at the brainstem level^21,22^. Prepulses are indeed ubiquitous. They are likely generated by inputs of exteroceptive and interoceptive origin, such as visual, auditory, somatosensory and other types of stimuli, integrating at subcortical level before impinging on the prepulse circuit to inhibit undesired reflex reactions, which would otherwise interfere with ongoing sensory processing^23^. We demonstrated that prepulse effects on the TBR vary according to body posture and to the site where prepulses are applied^24^.

Moreover, a PPI-like effect has been proposed for the inhibition of the R2 component of the TBR (TBR-R2) induced by self-stimulation; several pre-stimulus inputs may in fact attract a subject’s attention and, therefore, deviate it from the threatening situation^15^.

A possible interaction between PPI and peripersonal space of the face was investigated by Kiziltan et al.^25^, who reported reduced TBR-R2 inhibition by preceding finger stimulation when the hand was held near the face rather than far from it^25^.

There are no studies to date of the effects of prepulses on the HBR.

Here we aimed at investigating subcortical modulation of the HBR exerted by prepulse stimulation and its interaction with its well-known cortical (cognitive) modulation.

We hypothesized that prepulses would inhibit the HBR similar to TBR-R2. In particular, we investigated whether PPI effects would overrule the enhancement of HBR elicited near the face due to facilitatory modulation by the parieto-frontal, multisensory-motor system involved in representation and interaction with the space that closely surrounds the body (the peripersonal space)^26^. Furthermore, we aimed at assessing a possible difference in PPI induced by prepulses delivered either far from or near the participants’ face. Finally, we investigated whether self-elicitation of HBRs would also produce HBR response suppression, similar to PPI.

Results of the studies on HBR modulation by prepulses, their location relative to the face, and by self-stimulation, should expand our knowledge of the cortico-subcortical circuitries involved in behavioural changes of protective blinking and their interaction.

## Methods

All experimental procedures in this study were approved by the Institutional Review Board of Vipiteno Hospital. All methods and experiments were performed in accordance with the principles established by the Helsinki Declaration. Written informed consent was obtained from each subject prior to their participation in the experiment.

### Participants

Twenty healthy volunteers were recruited for this study, of whom six did not present with a clear and reproducible HBR despite stimulus intensities exceeding 60 mA delivered to the median nerve. The remaining fourteen healthy volunteers (8 females, age 32.6, SD 7.7 years; 11 right-handed) underwent one experimental session of HBR recordings with five different conditions.

### Stimulation and recording

All data were obtained with routine electrodiagnostic equipment (Viking EDX, Natus, Middleton, WI, USA). HBRs were elicited by electrical stimuli (constant current square-wave pulses of 0.2 ms duration) delivered to the median nerve of the dominant hand (“HBR-hand”) with a bar electrode, cathode proximal, which was firmly attached with a gauze pad and patch at the volar aspect of the wrist overlying the median nerve.

For applying electrical prepulses (constant current square-wave pulses of 0.2 ms duration, 2 times sensory threshold, or less in case of inadvertently eliciting overt blink reflexes), ring electrodes were attached to the proximal and middle phalanx of the non-dominant index finger (“prepulse-hand”).

First, we established sensory threshold for the non-dominant index finger. Sensory threshold was defined as the minimum stimulus intensity perceived in at least 4 out of 8 consecutive stimuli. Then we established the intensity necessary to elicit reproducible HBRs. To that, we applied stimuli to the dominant median nerve in increments of 10 mA until we obtained a response from OOc with more than 50 µV baseline-to-peak amplitude; subsequently we increased the intensity by 20 mA.

Single sweeps of electromyographic activity in OOc were recorded bilaterally with self-adhesive disposable electrodes attached to the skin, the active electrode overlying the middle portion of the muscle below the pupil when looking straight ahead, and the reference electrode lateral to the outer canthus. The electromyographic signal was amplified (× 1000), band-pass filtered (30–3000 Hz), and rectified.

In the first four conditions (see below) all stimuli were delivered by the examiner, whereas in the last condition participants triggered the stimuli themselves. Subsequent stimuli were separated by at least a 30 s interval in order to prevent habituation^5^.

### Procedures

Participants lay supine on a stretcher in a silent room at a comfortable room temperature and were asked to keep their facial muscles relaxed, keep their eyes open, and fixate a point on the ceiling. They were informed that the study would involve electric shocks delivered to their dominant hand and to the contralateral index finger, without providing further details about the different experimental procedures. Five experimental conditions were tested in blocks, which were pseudo-randomized across participants.

**Figure 1:**
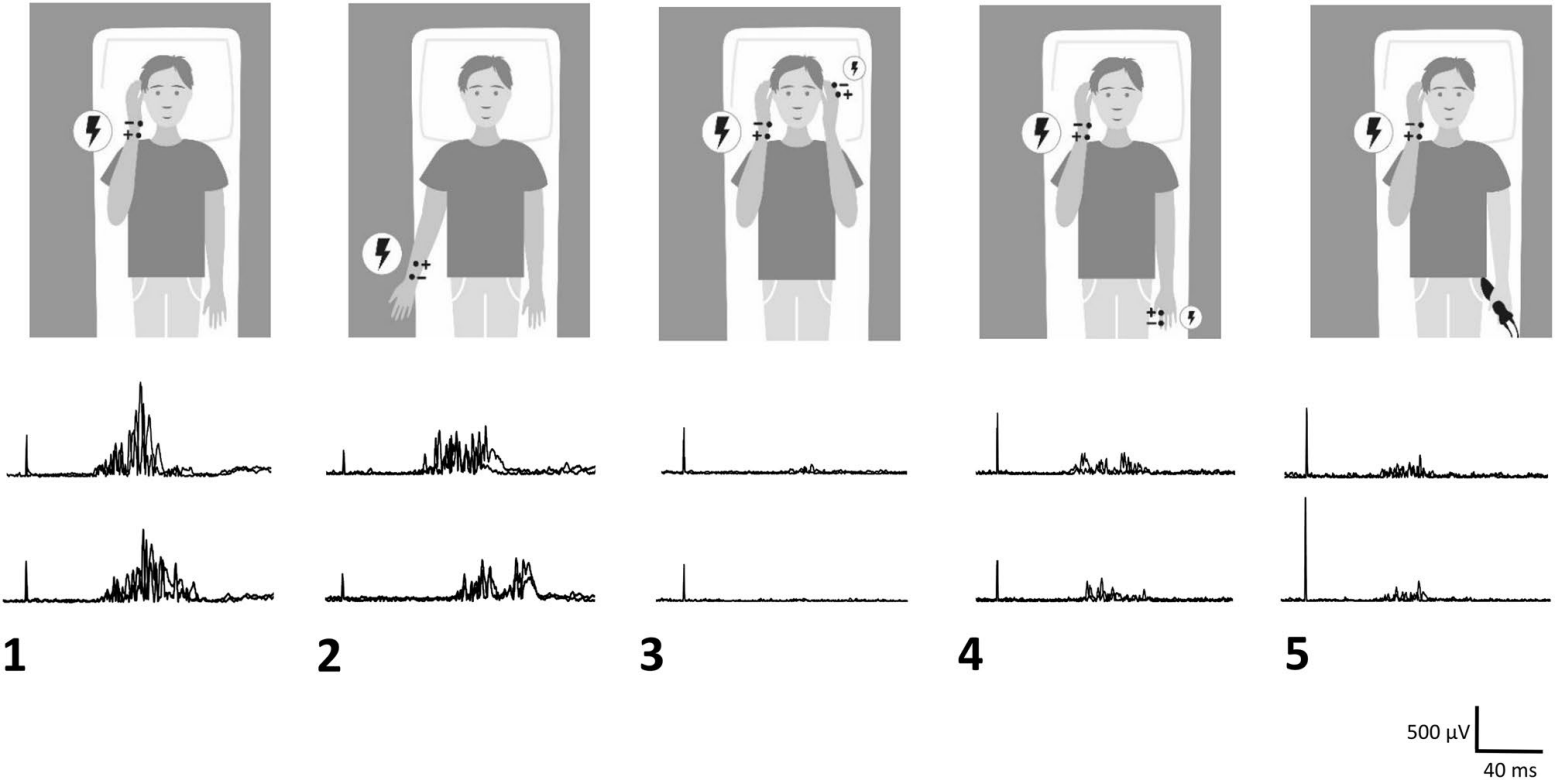
Representative examples of HBR recordings in a healthy participant in experimental *conditions 1–5*. In *conditions 1–4*, an experimenter triggers the stimuli; in *condition 5*, the participant triggers stimuli herself. In *condition 1*, the participant flexes the elbow and brings the dominant HBR-hand near to the face; the median nerve is stimulated at the wrist. In *condition 2*, both arms are outstretched at the side of the participant’s body; the median nerve is stimulated at the wrist of the dominant HBR hand. In both *conditions 1 and 2*, there is no prepulse, while in both *conditions 3 and 4*, prepulses are delivered to the non-dominant index finger 100 ms preceding median nerve stimulation to the HBR-hand. In *condition 3*, both HBR-hand and prepulse-hand are held close to the face. In *condition 4*, the HBR-hand is held near to the face, while the non-dominant prepulse-hand is outstretched. In *condition 5*, the HBR-hand is held near to the face, while the participant triggers with the non-dominant hand HBR-eliciting stimuli to the dominant median nerve (see text for more detailed explanations). There is no prepulse in *condition 5*. Two superimposed rectified HBR recordings are presented for each condition, showing responses in orbicularis oculi muscle ipsilateral (top traces) and contralateral (bottom traces) to median nerve stimulation. Note that contralateral HBR responses tend to be smaller with longer onset latencies than recordings obtained ipsilateral to median nerve stimulation. The hand-to-face proximity in *condition 1* induces, as compared to *condition 2*, a facilitation of HBRs, i.e., increase of area and shortening of latency*.* In *conditions 3 and 4*, HBRs are profoundly inhibited, i.e., area is markedly reduced, and onset latency is lengthened, with more inhibition present in *condition 3* compared to *condition 4*. In *condition 5*, self-stimulation produces a reduction in HBR area but also a shortening of the onset latency.

In *condition 1 (no-prepulse-HBR-near*), HBRs were elicited in the participants lying supine with the elbow flexed so as to hold the HBR-hand at about 10 cm in front of the participant’s face, while the other hand remained outstretched lying at the side of the participant’s body; this represented our baseline condition against which all other conditions were compared (Fig. 1).

In *condition 2 (no-prepulse-HBR far*), HBRs were obtained with both hands resting at the side of the body; the median nerve of the HBR-hand was then stimulated in the “far” position with respect to the face (Fig. 1).

In *condition 3 (prepulse-near-HBR-near*), HBRs were elicited with the HBR-hand positioned near the face, but now a prepulse was delivered to the contralateral index finger (“prepulse-hand”) 100 ms before the median nerve stimulus to the HBR-hand; the prepulse-hand was also brought close to the participant’s face (Fig. 1).

In *condition 4 (prepulse-far-HBR-far*), HBRs were elicited again with the HBR-hand held near the face, yet again a prepulse was delivered to the index finger 100 ms prior; however, the prepulse-hand remained outstretched lying at the side of the participant’s body (Fig. 1).

In *conditions 1–4*, an experimenter elicited HBRs by pressing a button on the console of the electrodiagnostic machine without the participant’s knowing when and where stimulation would occur. We took special care that the act of triggering remained unnoticed by the participants, as they may be warned that the stimulus is about to arrive by some characteristic movement of the examiner, or any other cue just before reflex elicitation, which may have an effect on reflex characteristics^15^. The action of pressing the trigger button did not produce noise (beep turned off)^15^.

Five responses were obtained in each condition with an inter-trial interval of at least 30 s, in order not to produce response habituation over time^5^. Stimuli were repeated online whenever necessary, e.g., when a spontaneous blink interfered with the recordings, or when background electromyographic (EMG) activity was detected by visual or acoustic feedback.

Finally, HBRs were elicited again with the HBR-hand held near the face, but this time the participants were given the trigger device into their outstretched contralateral hand. They were asked to deliver the stimuli at their own pace, after being informed each time when an inter-trial interval of 30 s had passed by (*condition 5—self-stimulation-HBR-near*) (Fig. 1); again, recordings were discarded in case of contamination with spontaneous blinks or too much background EMG activity.

We decided to deliver sensory prepulses not to the dominant hand where also the median nerve stimulation took place, but to the contralateral hand, thereby allowing us to manipulate the prepulse-hand position while keeping the HBR-hand close to the face.

### Data analysis and statistics

We determined latency and area-under-the-curve (henceforth “area”) of the HBR in OOc ipsi- and contralateral to the stimulated median nerve. HBR onset latency was measured in each single rectified trace by manually placing a cursor when the EMG recording exceeded background activity by 30 µV within a measurement window of 30–110 ms. End latency was determined by manually placing a cursor when EMG activity dropped back to baseline. The EMG machine produced the area-under-the-curve value between the two markers (sum of rectified voltage values times sampling interval). We then averaged the raw data per participant for each of the conditions.

In addition, we measured the EMG area in OOc bilaterally during a 100 ms time window preceding the stimulus in all conditions, to rule out differences in background EMG activity, e.g., due to squinting of eyes.

Subsequently, in order to compare HBRs in different test conditions, we normalized all data: we arbitrarily assigned 100% to the median latency and area values obtained in the OOc on the side of median nerve stimulation for each participant in *condition 1* and represented all individual values in all 5 conditions as respective percentages. We used median values to avoid possible skew in case of outliers. These relative values were then averaged per participant and per condition, to calculate group means and standard deviations for latency and area of HBRs.

Normal data distribution of all absolute and percentage values was confirmed within each condition with Kolmogorov–Smirnov testing.

Pre-stimulus baseline EMG was compared for each OOc separately across all 5 conditions applying repeated measures ANOVA (factor “condition”: five levels). Absolute mean values of OOc recordings (HBR latency, HBR area) were compared between the ipsi- and contralateral side to the stimulated median nerve applying two-factor repeated measures ANOVA (factor “side”: two levels; factor “condition”: five levels), followed up by paired Student’s t-tests in each condition.

Comparative statistics of mean percentage values was performed to assess whether different study conditions exerted an effect on the HBR applying repeated measures ANOVA (factor “condition”: five levels). Paired t-tests were used in post-hoc analyses to follow-up on significant differences.

For all ANOVAs, sphericity of data was tested according to Mauchly, and when < 0.05 appropriate correction was applied according to Greenhouse–Geisser.

The level of significance was set at *p* < 0.05 for all comparisons with correction for multiple testing by false discovery rate according to Benjamini-Hochfeld.

## Results

We observed a clear and reproducible HBR in 14 out of 20 participants (70%), who completed the study without difficulty. In those 14 participants who showed a response, HBRs were obtained to each median nerve stimulus in the HBR-hand in all five experimental conditions throughout the whole experiment.

Mean sensory threshold (± standard deviation) for non-dominant index finger stimulation was 1.8 ± 0.4 mA. Effective stimulus intensity for index finger stimulation (not eliciting any overt blink reflexes) was 3.2 ± 0.6 mA. Mean sensory threshold for median nerve stimulation at the wrist was 2.0 ± 0.9 mA. Threshold intensity for eliciting HBRs was 14.6 ± 11.0 mA. Effective stimulus intensity used for median nerve stimulation to test HBRs was 33.1 ± 11.1 mA.

Absolute HBR parameters obtained in each condition are listed in Table 1. All HBR parameters were normally distributed. Recording side tended to have a significant main effect on HBR latency (F_1,13_ = 4.600, *P* = 0.051, *η*_*p*_^*2*^ = 0.261) without significant interaction of side × condition (F_1.922,24.983_ = 0.801, *P* = 0.455, *η*_*p*_^*2*^ = 0.058) while having no significant main effect on HBR area (F_1,13_ = 2.479, *P* = 0.139, *η*_*p*_^*2*^ = 0.095) and no interaction of side × condition (F_1.808,23.503_ = 1.365, *P* = 0.273, *η*_*p*_^*2*^ = 0.095). In all conditions, the HBR tended to be larger on the side ipsilateral to the stimulated median nerve, reaching statistical significance only in *condition 2* (*no-prepulse-HBR-far)* (*P* = 0.030), while HBR latencies were significantly shorter on the side ipsilateral to median nerve stimulation in *prepulse and self-stimulation conditions* (Table 1).

**Table 1:** Hand-blink reflex parameters for recordings ipsi- and contralateral (HBR_i_, HBR_c_) to median nerve stimulation.

	Condition	HBR_i_ latency [ms]	HBR_c_ latency [ms]	*P*-values	HBR_i_ area [µVms]	HBR_c_ area [µVms]	*P*-values
1	No-prepulse-HBR-near	42.2 (1.7)	43.5 (1.4)	0.098	1663 (217)	1522 (202)	0.326
2	No-prepulse-HBR-far	45.4 (1.5)	46.1 (1.3)	0.377	1192 (200)	986 (154)	**0.030**
3	Prepulse-near-HBR-near	48.3 (2.2)	49.0 (2.2)	**0.017**	385 (58)	340 (47)	0.331
4	Prepulse-far-HBR-near	45.8 (2.0)	47.2 (1.7)	**0.047**	517 (75)	473 (53)	0.506
5	Self-stimulation-HBR-near	42.1 (2.0)	43.3 (1.7)	**0.022**	537 (58)	483 (59)	0.303

Figures are the mean values (standard error within parenthesis) for group average data of individual absolute mean values. *P*-values indicate differences between HBR_i_ and HBR_c_ (paired t-tests).

Absolute prestimulus EMG values (assessed during 100 ms preceding median nerve stimulation) did not differ significantly among conditions on either side (ipsilateral OOc: F_4,52_ = 0.451, *P* = 0.772, *η*_*p*_^*2*^ = 0.033; contralateral OOc: F_2.056,26.727_ = 0.318, *P* = 0.736, *η*_*p*_^*2*^ = 0.024).

Comparison of relative HBR values among the various conditions revealed a main effect for HBR latency (ipsilateral: F_2.036,26.474_ = 5.314, *P* = 0.011, *η*_*p*_^*2*^ = 0.290; contralateral: F_2.174,28.263_ = 4.736, *P* = 0.015, *η*_*p*_^*2*^ = 0.267) and for HBR area (ipsilateral: F_4,52_ = 52.885, *P* = 0.000, *η*_*p*_^*2*^ = 0.803; contralateral: F_2.523,32.802_ = 42.763, *P* = 0.000, *η*_*p*_^*2*^ = 0.767). Compared to *condition 1,* post-hoc pairwise comparisons revealed significantly longer latencies in *condition 2* (i.e., *HBR-far* vs *HBR-near*), and in both *conditions 3 and 4* (i.e., *prepulse-near-HBR-near* and *prepulse-far-HBR-near* vs *no-prepulse-HBR-near*, respectively). HBR latencies following *self-stimulation* were significantly shorter compared to both *prepulse conditions* but not compared to *condition 1* (Table 2).

**Table 2:** Hand-blink reflex (HBR) parameters.

	Condition		Relative HBR_i_ latency	Relative HBR_c_ latency	Relative HBR_i_ area	Relative HBR_c_ area
1	No-prepulse-HBR-near	Mean (SE)	100.9 (0.8)	104.7 (2.5)	102.2 (2.8)	101.2 (9.0)
2	No-prepulse-HBR-far	Mean (SE)	109.2 (2.3)	111.6 (4.0)	75.6 (7.8)	69.6 (9.8)
3	Prepulse-near-HBR-near	Mean (SE)	117.2 (6.3)	118.9 (6.4)	29.7 (5.6)	27.4 (4.6)
4	Prepulse-far-HBR-near	Mean (SE)	110.1 (3.9)	114.1 (4.8)	38.8 (6.5)	38.8 (6.8)
5	Self-stimulation-HBR-near	Mean (SE)	101.2 (3.4)	104.5 (3.5)	40.2 (5.4)	43.0 (10.3)
	1 vs 2	*P*	**0.002**	**0.010**	**0.002**	**0.000**
	1 vs 3	*P*	**0.023**	**0.032**	**0.000**	**0.000**
	1 vs 4	*P*	0.**029**	**0.018**	**0.000**	**0.000**
	1 vs 5	*P*	0.939	0.941	**0.000**	**0.000**
	2 vs 3	*P*	0.166	0.186	**0.000**	**0.000**
	2 vs 4	*P*	0.756	0.354	**0.000**	**0.000**
	2 vs 5	*P*	0.038	0.043	**0.000**	**0.003**
	3 vs 4	*P*	0.114	0.332	**0.015**	**0.003**
	3 vs 5	*P*	**0.007**	**0.009**	**0.041**	0.056
	4 vs 5	*P*	**0.022**	**0.024**	0.716	0.408

Figures are mean values (standard error, SE) for group average data in percentage of individual median values of the orbicularis oculi muscle ipsilateral to the stimulated dominant median nerve in *condition 1* (*no-prepulse-HBR-near*). *P* values (paired t-tests) indicating statistically significant differences following Benjamini–Hochberg procedure to compensate for multiple testing are in bold.

*HBR*_*i*_ ipsilateral, *HBR*_*c*_ contralateral.

**Figure 2:**
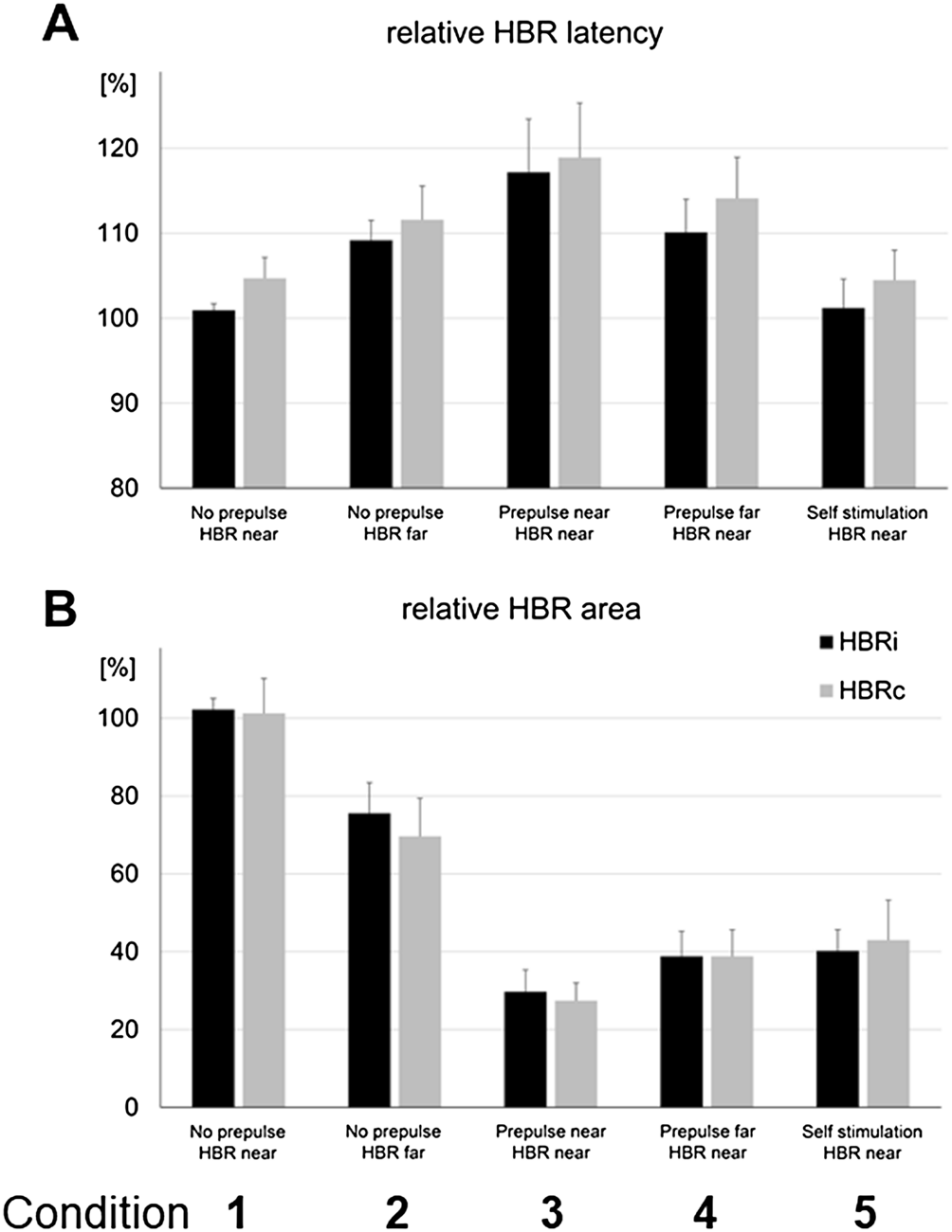
The columns represent normalized group data for onset latency (**A**) and area (**B**) of HBRs recorded in orbicularis oculi muscle ipsi- (black columns) and contralateral (gray columns) to dominant median nerve stimulation in experimental *conditions 1–5* (see text for details). The columns are mean values (whiskers are mean standard errors) for group average data in percentage of individual median values of the orbicularis oculi muscle ipsilateral to the stimulated dominant median nerve in *condition 1* (*no-prepulse-HBR-near*).

HBR area decreased significantly in *condition 2* relative to *condition 1* (i.e., *HBR-far* relative to *HBR-near*), and also in *conditions 3* and *4* relative to *conditions 1* and *2* (i.e., a prepulse suppressed HBR). Notably when the prepulse was delivered with the hand close to the face (*condition 3*), the HBR was suppressed even more profoundly than when the hand was outstretched next to the body (*condition 4*). Self-stimulation (*condition 5*) also suppressed the HBR bilaterally similar to prepulses delivered to the index finger held far from the face (*condition 3*), but to a lesser degree than when delivered near the face (*condition 4*) (Figs. 1, 2; Table 2).

## Discussion

In the present study, we describe for the first time PPI of the HBR, which adds to the already described PPI effects on trigeminal and auditory blink reflexes and on the auditory startle reaction^15,19,24,27^. Importantly, we have demonstrated that such PPI occurs on HBR responses that are facilitated by holding the stimulated hand near the face. Thus, the attentional shift caused by prepulse stimuli was capable of overruling the profound augmentation of the HBR near the face, which reflects the increased behavioural relevance of blinking in response to threat in a self-centred frame of reference^28^. Furthermore, the amount of PPI was influenced by the proximity of prepulses to the face. In fact, HBRs, facilitated by holding the HBR-hand near the face, were even more profoundly suppressed when also the prepulse-hand was held near the participant’s face, as compared to the amount of PPI when the prepulse-hand was outstretched at the side of their body.

Self-stimulation of HBR generated a significant inhibition of the response, too, but to a lesser extent compared to that induced by prepulse stimulation.

### Confirmatory results: the HBR is facilitated when the stimulated hand is near the face

We chose *condition 1* (*no-prepulse-HBR-near*) as reference condition because each other condition differed in only one experimental factor, i.e., either position of the hand (far vs near, in *conditions 1* and *2*), presence of a prepulse on the contralateral hand (*conditions 3* and *4*), or agency (stimulation by experimenter vs self-stimulation, *condition 5*).

Compared to *condition 2 (no-prepulse-HBR-far)*, we found a significantly enlarged HBR area and shortened HBR latency in *condition 1 (no-prepulse-HBR-near)*. HBR area increased by some 50% (Table 2), and HBR latency shortened on both sides by some 3 ms, when the hand was brought close to the face (Table 1).

The effect of hand position on HBR concurs with previous literature^5,6^. These authors have reported that stimuli to the hand held near the face induced a top-down facilitation of brainstem interneurons that ultimately results in stronger protective blinking. Interneurons in the reticular formation play a crucial role in influencing the magnitude of the eyeblink reflex as their excitability can be rapidly pre-set via descendent projections from higher-order areas^23^.

Several physiological studies in humans support the existence of a “defensive peripersonal space network” involving the ventral premotor and the posterior parietal cortices, dedicated to special cross-modal (tactile, auditory, and visual) representation of the space directly surrounding the body, to detection of potential threats, and to rapid preparation of appropriate motor reactions (among others:^29–35^).

Specifically, the HBR-derived ‘defensive peripersonal space’ is a response field around the face where the evoked HBR gradually increases inversely to stimulus distance to the face, starting from a critical distance, within which the nervous system deems it appropriate to enhance motor behaviour aimed at self-protection^10^. A wealth of other stimulus-related or unrelated factors modulate HBR magnitude, with the overall aim to increase the behavioural utility of this response. These factors are e.g. stimulus direction and speed of movement, body position in space and with respect to stimulus (gravitational attraction), body motion, biological stimulus valence, environmental scenario with the presence of possible additional unfavourable or protective factors, and state of the subject, i.e. level of mental and physical resources to cope with the menace^10^. However, the spatial proximity between the stimulus and the face remains a key factor for HBR facilitation. Proximity of danger is likely the main factor triggering a fight or flight response.

Similarly to Sambo et al.^5^, we found significantly shorter HBR onset latencies, and significantly larger HBR areas, in the OOc muscle ipsilateral to median nerve stimulation compared to the contralateral one. We consider these interside differences indeed plausible, as they are also present for, e.g., the TBR^27,36,37^ due to the longer, polysynaptic, intrinsic brainstem pathways that mediate responses in the contralateral OOc.

### Prepulse inhibition of the HBR and its spatial modulation

In *condition 3 (prepulse-near-HBR-near)* and *condition 4 (prepulse-far-HBR-near),* we observed a profound suppression of HBRs when a peri-liminal electrical stimulus (at 2 times sensory threshold intensity) was applied to the non-dominant index finger 100 ms preceding the strong reflex-eliciting stimulus to the median nerve on the dominant side.

The mean residual HBR was < 30% of unconditioned responses in *condition 3* and < 40% in *condition 4* (Table 2).

PPI of the HBR is an expected finding given the physiological analogy of the HBR with other protective brainstem reflexes such as the TBR or the auditory startle reaction^23,27^, that are inhibited by prepulses to a similar extent.

The proposed mechanism underlying PPI is considered to be an attentional shift towards the sensory input brought about by the prepulse^16,38^. Thus, PPI reflects an early stage of attentional processing pertaining to information selection that operates at the subcortical level outside of conscious awareness^23,39^.

PPI is a very robust phenomenon, which can be observed across different sensory systems in numerous species. PPI mediating areas are located within the brainstem and include caudal pontine reticular nucleus and pedunculopontine nucleus (for review see^21^).

Several findings suggest the existence of a cortico-limbic modulatory network, including nucleus accumbens, basolateral amygdala, septohippocampal system, and medial prefrontal cortex^40^ that continuously adjusts PPI in relation to attentional and emotional states^41^. Inhibition plays a crucial role in many functional domains, such as cognition, emotion, and motor control. A reduction of PPI is thought to reflect dysfunction of sensorimotor gating which normally suppresses excessive behavioural responses to intrusive stimuli.

As demonstrated by the present findings, PPI of the HBR can be modulated by changing arm position, thus altering the physical distance between the prepulse-hand and the face, as it happens for HBR itself. PPI of the HBR was significantly more pronounced, i.e. the HBR was more suppressed, when the prepulse-hand was held near the face rather than far from it, while always keeping the HBR-hand in the “threatening” position near the face.

An influence of changing the position of the prepulse-hand relative to the face has recently been investigated for the TBR by Kiziltan et al.^25^. They reported reduced PPI of TBR-R2 when the index finger, to which prepulses were delivered 100 ms before supraorbital nerve stimulation, was positioned near the face rather than far from it^25^. Possibly, these seemingly contradicting results might be due to functional–anatomical differences in sensorimotor circuits that underlie TBR and HBR, the former including the pontine reticular formation, the latter involving the mesencephalic reticular formation^42^. In addition, experimental methodology differs: based on typical onset latencies of HBR and TBR, the afferent conduction time for the reflex triggering stimulus to reach the relevant brainstem structures may differ by some 20 ms, respectively. Perhaps different ISI may result in different reflex modulation, and a longer ISI, e.g. 120 ms between prepulse and pulse, might have caused similar HBR reflex modulation as reported for the TBR by Kiziltan et al.^25^.

Our findings of an increased PPI, when a prepulse occurs near the face, opens up several possible interpretations.

Stimulus location-related PPI enhancement might occur entirely at the subcortical level due to augmented proprioceptive input, when the prepulse-hand is held near the face, thus with the arm tonically flexed at the elbow and elevated against gravity, as opposed to a resting position beside the body. A possible influence of disparate proprioceptive input on PPI has previously been demonstrated for upper and lower limb sensory input while standing compared to lying supine^24^. At any rate, PPI is considered a strictly subcortical mechanism based on pre-synaptic inhibition between sensory inputs^43^.

Although speculative, PPI might be—similarly to HBR—modulated in a top-down manner via descending projections from the above mentioned “defensive peripersonal space network”. Thus, both pulse and prepulse stimuli would be interpreted by higher order areas as being biologically more relevant when delivered in close proximity to the face. As a consequence, the ensuing inhibitory effect on the HBR is enhanced. Intriguing as they are, the present data do not allow such interpretation to a full extent, as additional experiments would be required for further elaboration and confirmation, e.g. additional prepulse-hand and HBR-hand locations, further prepulse modalities, and other protective reflexes. An important question remains: what is the biological advantage of an increased PPI at the expense of protective eye-closure? Possibly, the defence of the body from an imminent threat within the peripersonal space may in certain situations depend on keeping the eyes open rather than closing and protecting the eyes from possible injury. E.g., a person driving a vehicle has a clear survival advantage if they keep their eyes open despite a sudden and threatening stimulus to the eyes.

### Self-stimulation inhibits HBR

HBR area is suppressed by self-stimulation compared to both *no-prepulse-conditions*, is similar to *condition 4* (*prepulse-far-HBR-near*) but is less suppressed than with prepulses delivered near the face (Fig. 2B, Table 2). Furthermore, HBR latency is shortened by self-stimulation in comparison to both *prepulse conditions*, but not as compared to conditions without prepulses, in particular to our reference *condition 1* (*no-prepulse-HBR-near*) (Fig. 2A, Table 2).

It is generally accepted that HBRs are facilitated with respect to magnitude and latency when elicited near the face^5^. Considering the similarity of HBR latencies in *conditions 1 and 5*, we can assume that self-stimulation might in fact also have a facilitatory effect on HBR latencies, despite concomitant suppression of HBR area, at least relative to HBRs elicited far from the face. This is principally similar to the pattern observed for TBR-R2, which shows longer latencies with PPI^27^, but not with self-stimulation^15^, whereas R2 area is suppressed by both^15,24,27^. These observations suggest that the effect of self-stimulation is rather not due to a PPI effect, but perhaps to self-agency.

The effect of self-stimulation on the TBR has long been acknowledged^44–46^. In a recent and detailed study, different possible mechanisms for the observed suppression of TBR-R2 have been put forward, being either due to an attention-shift phenomenon, or due to sensory attenuation^15^.

The basic mechanisms of reflex modulation exerted by self-stimulation may pertain to both TBR and HBR, therefore they are summarized here again (for details see^15^). Brain responses are smaller^47^ and perception is less intense in response to self-produced events. The physiological purpose of this phenomenon, termed “sensory attenuation”^48^, is to identify self-produced effects and to distinguish them from externally produced ones. A commonly accepted explanation suggests that internal forward models involving the supplementary motor area^48,49^, entail an efferent copy of the motor command, which simulates the executed movement in advance, and which is used to predict its consequences^50^. Sensory attenuation and self-agency seem to be strongly linked^51^. Volitional activity, sensory events, and motivational and emotional factors on reflexes have long been known to influence the excitability of reflex pathways in polysensory integrative brainstem centres^52^. Therefore, also reflex responses may differ depending on whether they are self-generated or externally elicited by another person. The act of self-eliciting a stimulus produces an efferent copy of the motor command, which may not only lead to sensory attenuation but may possibly also inhibit reflex responses. When perturbations are unexpected, ensuing reflex responses may be crucial for an individual’s health and survival, but when such perturbations are predictable (e.g., when stimuli are self-induced), the same reflex responses bear less functional benefit and hence are suppressed.

### Limitations

We cannot exclude that the HBR modulation observed in *condition 3* (*prepulse-near-HBR-near*) could be due to the proximity of the “prepulse hand” to the face irrespective of actual prepulse delivery. Moreover, the suppressive effect on HBR may also arise from the mere proximity of both stimuli to each other (the reflex-eliciting stimulus and the prepulse), rather than the proximity of the prepulse to the face. Future experiments may serve to clarify these aspects.

Finally, we did not test an experimental condition in which the HBR-hand is far from the face while a prepulse is given to the contralateral hand. We decided to assess PPI in the *HBR-near condition* in which maximum HBR magnitude is expected.

### Conclusion

The reactivity of brainstem neurons and circuits mediating blink reflex activity to sensory inputs is under both excitatory and inhibitory control from rostral areas. Blinking stronger when environmental stimuli have increased potential to harm the eye has a clear survival advantage; however, reducing blinking or even suppressing it could in certain situations be convenient in order not to interrupt ongoing sensory processing. Preceding peri-liminal prepulses could be the underlying physiological mechanism serving as warning signals.

## Data Availability

The data that support the findings of this study are available on request from the corresponding author.
